# Large–Scale Movement and Reef Fidelity of Grey Reef Sharks

**DOI:** 10.1371/journal.pone.0009650

**Published:** 2010-03-10

**Authors:** Michelle R. Heupel, Colin A. Simpfendorfer, Richard Fitzpatrick

**Affiliations:** 1 School of Earth and Environmental Sciences, James Cook University, Townsville, Queensland, Australia; 2 Fishing and Fisheries Research Centre, School of Earth and Environmental Sciences, James Cook University, Townsville, Queensland, Australia; 3 The Reef Channel, Cairns, Queensland, Australia; Northern Fisheries Centre, Australia

## Abstract

Despite an Indo-Pacific wide distribution, the movement patterns of grey reef sharks (*Carcharhinus amblyrhynchos*) and fidelity to individual reef platforms has gone largely unstudied. Their wide distribution implies that some individuals have dispersed throughout tropical waters of the Indo-Pacific, but data on large-scale movements do not exist. We present data from nine *C. amblyrhynchos* monitored within the Great Barrier Reef and Coral Sea off the coast of Australia. Shark presence and movements were monitored via an array of acoustic receivers for a period of six months in 2008. During the course of this monitoring few individuals showed fidelity to an individual reef suggesting that current protective areas have limited utility for this species. One individual undertook a large-scale movement (134 km) between the Coral Sea and Great Barrier Reef, providing the first evidence of direct linkage of *C. amblyrhynchos* populations between these two regions. Results indicate limited reef fidelity and evidence of large-scale movements within northern Australian waters.

## Introduction

Conservation concerns about the status of coral reef associated shark populations in various locations around the world [1]–[4] make it increasingly important to understand the movements and ecology of these species. Randall [5] suggested that since reef sharks are widely distributed throughout the Indo-Pacific they must make large-scale movements at some points (although these events may be rare). This suggests potentially complex patterns where some individuals are site attached while others make large-scale dispersal movements, but there are no direct data for most species.

Defining movement patterns within reef shark populations is vital to interpreting results of sampling such as catch data and underwater visual surveys that have been used to infer reductions in reef shark populations. Recent studies have revealed higher relative abundances of reef sharks in areas closed to fishing and public entry [1]–[2], [4]. These results suggest that area closures are supporting larger populations of site attached reef sharks, while fished regions have been locally depleted. Understanding the movement patterns and level of site attachment in reef sharks is crucial to interpreting these results and designing effective future management (e.g. closed areas) for reef shark populations. Here we present data on movement patterns of *Carcharhinus amblyrhynchos* from the Great Barrier Reef, Australia. The aims of this study were to: 1) determine the level of fidelity to individual reef platforms and marine protected areas, 2) examine the extent of movement within a series of closely associated reef platforms, and 3) determine if there are ontogenetic differences in fidelity and movement patterns.

## Results

**Figure 1:**
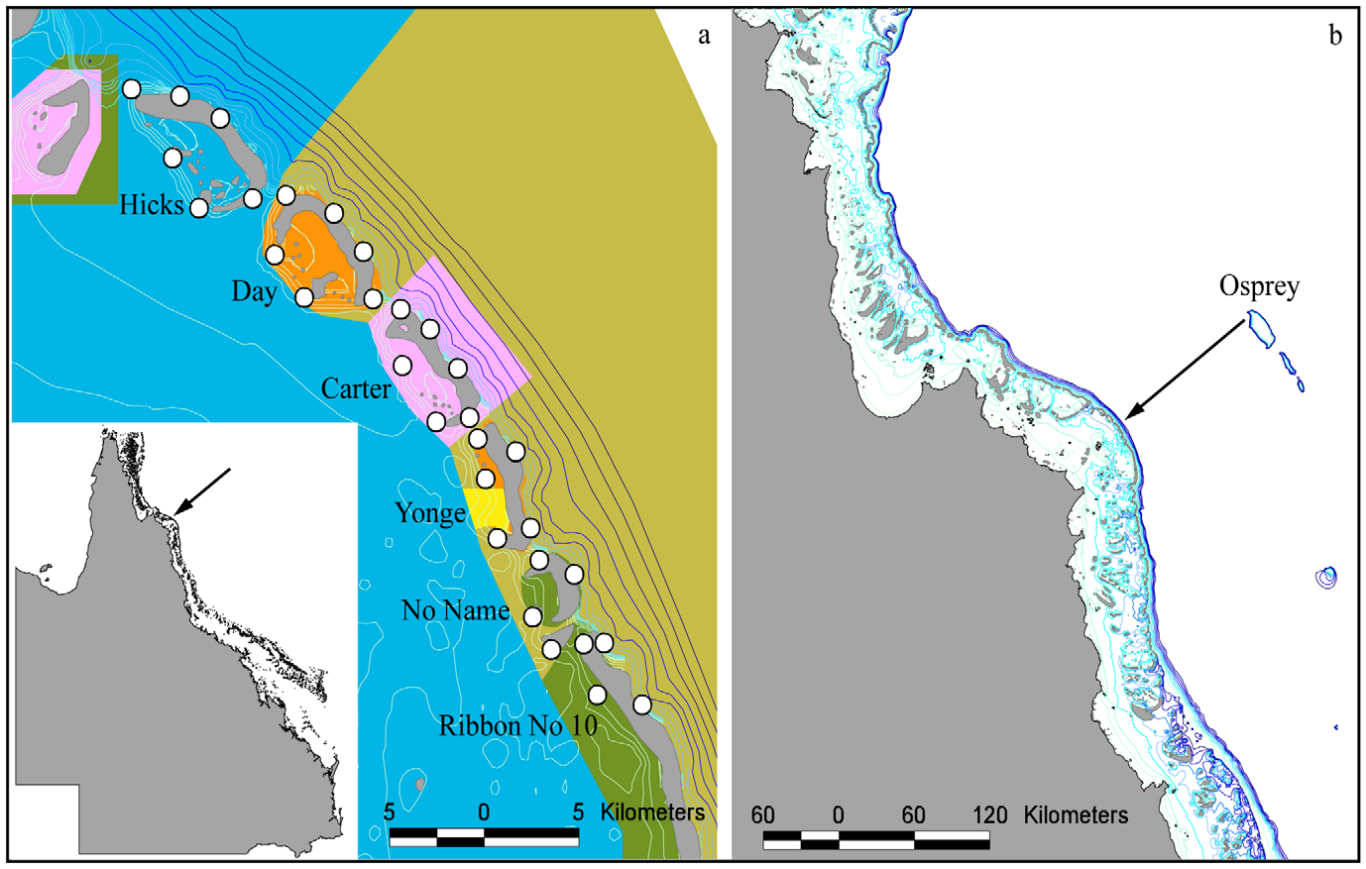
Acoustic monitoring array location and indication of observed large-scale open ocean movement. a) Monitoring array within the Ribbon Reefs. White points indicate receiver locations. Colored polygons indicate reef zonation where blue  =  Habitat Protection Zone, yellow  =  Conservation Park Zone, orange  =  Scientific Research Zone, green  =  Marine National Park Zone, pink  =  Preservation Zone and tan  =  Buffer Zone. Inset (arrow) indicates location along the Queensland coast. b) Movement of 122 cm TL male from Osprey to the Ribbon Reefs. Lines in both panels represent depth contours.

In February 2008 nine *C. amblyrhynchos* ranging from 84–152 cm total length (TL) were fitted with acoustic transmitters in the Ribbon Reefs (Figure 1) including four males and five females (Table 1). One individual (7929) was not detected on the acoustic array after release and was not considered in any analyses. All but one of the remaining individuals left the monitored region during the study period with the maximum number of days detected being 130. There was no consistent pattern in whether individuals were detected more during the day than at night. Of the sharks detected 10 or more times, two were detected significantly more during the day, one was detected significantly more at night and three did not have any significant differences between day and night (Table 1). Three patterns of movement were observed: 1) presence at a single reef, 2) movement between and among reef platforms, and 3) movement away from the monitored area.

**Table 1:** *Carcharhinus amblyrhynchos* monitored at the Ribbon Reefs. Proportion of days monitored/days detected (in brackets).

Transmitter number	Total length (cm)	Sex	Release date	Number of days monitored	Number of days detected	Day time detections	Night time detections	X^2^	p
7921	108	M	6/2/2008	13	10 (76.9%)	412	72	237.4	<0.001
7924	117	M	6/2/2008	154	1 (0.6%)				
7925	124	F	6/2/2008	33	3 (9.1%)	7	3	0.9	0.343
7927	152	M	6/2/2008	167	22 (13.2%)	69	60	0.5	0.481
7928	90	F	6/2/2008	72	5 (6.9%)	58	11	30.7	<0.001
7929	103	M	6/2/2008	0	0				
7930	152	F	6/2/2008	150	100 (73.3%)	513	873	94.0	<0.001
7940	84	F	5/2/2008	150	98 (65.3%)	450	408	2.0	0.162
7944	93	F	6/2/2008	148	130 (87.8%)	708	325	141.3	<0.001
7908*	122	M	27/3/2008	2	2 (100%)				

*individual released at Osprey Reef.

**Figure 2:**
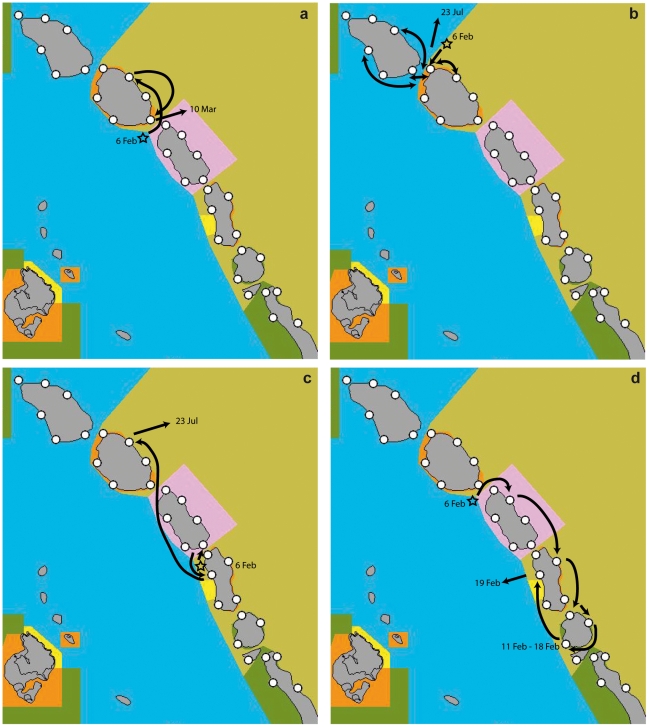
Movement of four *C. amblyrhynchos* within the Ribbon Reef monitoring array. a) Movement confined to a single reef, b) repeated movement between two adjacent reefs, and c–d) longer distance movement among reefs. Arrows indicate direction of movement, double headed arrows indicate repeated movement between locations. Colored polygons indicate reef zonation where blue  =  Habitat Protection Zone, yellow  =  Conservation Park Zone, orange  =  Scientific Research Zone, green  =  Marine National Park Zone, pink  =  Preservation Zone and tan  =  Buffer Zone.

Five of the eight sharks were only recorded at one reef and this was typically the reef where the individual was released. Two of these individuals (7940, 7928) were juveniles and some of the smallest individuals in the sample. These two individuals were recorded over long periods and showed significant reef fidelity. One of these individuals was released at Hicks Reef (zoned HP), recorded there on 98 of 150 days and was still present at the time of receiver removal. The second individual released at Yonge Reef (zoned SR and CP) covered a straight line distance of at least 5 km and was present at Day Reef (SR zone) from 6 Feb to 10 Mar before leaving the monitoring area (Figure 2a). The remaining three individuals recorded at a single reef were larger (7924, 7925, 7927) and two of the three were monitored for one and three days, respectively, before leaving the monitoring area. The third individual was heard over an extended period at a single receiver which may reflect mortality after release rather than restricted movement.

The remaining three sharks moved between reefs and protection zones. One individual moved between different protection zones up to 23 times, crossing a 1.5 km channel between reefs repeatedly with at least 15 movements from one reef to another (Figure 2b). This individual spent more time in the HP zone than the protective zones despite capture just outside the SR zone. The other two individuals changed reefs three to six times. One individual covered a straight line distance of >15 km over the course of five months (Figure 2c) and the other covered a straight line distance of >18 km in 14 days (Figure 2d). These individuals remained within protective zones, but regularly moved between reefs and showed limited fidelity to an individual reef. The proportion of time individuals that moved between reefs spent in protection zones was variable. One individual released in the HP (fished) zone was only recorded there 5% of the time, spending 95% of the monitored period in protective zones. The remaining two individuals were both released in MNP or SR zones with one individual spending 95% of its time in these zones and 5% of its time in the more restrictive P zone. The final individual spent 73% of its time within protective (SR and MNP) zones, 13% in the P zone and 13% in the fished HP zone.

One additional individual, a 122 cm TL male fitted with a transmitter at Osprey Reef in the Coral Sea (13° 54′ S, 146° 37′ E) was detected at the Ribbon Reef site. This individual was present at Osprey Reef from 27 Mar until 25 June 2008 and was next recorded on receivers at Day and Hicks Reefs starting 47.8 hours after the last detection at Osprey Reef. This is evidence that this individual travelled approximately 134 km across the open ocean from the Coral Sea to the Ribbon Reefs (Figure 1). This shark was recorded in the Ribbon Reefs for two days but was not detected further suggesting it left the monitoring area.

## Discussion

Individual *C. amblyrhynchos* monitored in the Ribbon Reefs showed variability in movement patterns. Smaller individuals appeared to be more site attached, while larger individuals were more likely to move beyond a single reef. This is similar to telemetry observations of *Negaprion brevirostris* and *C. perezi* in reef areas where juveniles showed high site fidelity to a small region [6]–[7]. Limited telemetry data exist for larger reef sharks, but data from the current study did not reveal high levels of fidelity with most individuals leaving the study area. Lack of detections/fidelity of larger individuals could have been the result of several factors. First, individuals could have been removed by fishermen. However, most individuals were released in protective zones and monitored within these areas, so removal through fishing is unlikely to have occurred. The presence of a small individual in the fished zone throughout the study period suggests fishing pressure on sharks in this region is low and is presumably even lower in protected areas. Estimates of higher abundance of sharks in protected zones [1]–[2] would also suggest removal from protected zones by fishers is unlikely. Another possible explanation is that the receiver coverage was not extensive enough to encompass movements of these individuals. However, if individuals were long-term residents moving around the reef complex additional detections would have been expected. The final, and most parsimonious, explanation is that larger individuals did not show high fidelity and moved out of the region.

Previous research in the GBR has revealed sharks are in higher abundance in areas closed to fishing [2], [4]. Such observations have been interpreted to indicate a relatively high degree of site fidelity at the level of individual reefs and that differences in abundance are the result of fishing. The current study has demonstrated that *C. amblyrhynchos* do show site fidelity to individual reefs, especially as juveniles. However, the lower level of site fidelity for larger individuals suggests that fishing may not be the only potential driver for differences in abundance. While the current study was not able to identify these drivers, potential mechanisms include differences in carrying capacity between fished and unfished reefs due to the ecosystem effects of fishing (e.g. reduce prey base on fished reefs) or behavioral differences between management zones that result in biased survey results

Research is beginning to reveal that as Randall [5] suggested, reef sharks disperse over large distances. Chapman *et al*. [8] reported movement of *C. perezi* over 50 km off the coast of Belize and the current study has demonstrated movement of 134 km for *C. amblyrhynchos*. A movement of 134 km in less than 48 hours required this individual to have swum at a rate of 0.6–0.7 body lengths per second, a rate of movement within those calculated for other shark species [9]–[10]. This result suggests connectivity between *C. amblyrhynchos* populations on Coral Sea Reefs and those of the Great Barrier Reef. Individuals do move large distances and these movements may be more common than previously thought.

Evidence of large-scale movements lends support to the hypothesis that undetected individuals in the Ribbon Reefs may have moved beyond the monitored region. Individuals showing fidelity over significant periods were juveniles, with adults using broader areas and moving out of detection range more quickly. Individuals could follow the chain of Ribbon Reefs north into the Torres Strait or south into the central Great Barrier Reef. None of the individuals fitted with transmitters in the Ribbon Reefs have been detected on receivers at Osprey Reef, but movement between these sites and of this magnitude can no longer be ruled out.

Based on this study it is apparent further research into the presence and movements of reef sharks is needed. Previous long-term studies of shark movement have been highly beneficial to understanding the ecology of those species and providing guidance for management. For example, studies of blacktip sharks (*C. limbatus*) in Florida have revealed detailed habitat use patterns and provided survival data useful to stock assessments and management decisions [11], [12]. Acoustic monitoring studies should be conducted over a longer time scale to determine how long individuals will remain present at reefs, if there are ontogenetic changes in site fidelity and if there are longer-term patterns of philopatry. There is also a need to examine inter-reef movements in regions where reefs are more widely spaced. The proximity of reef platforms in the Ribbon Reef complex may provide an opportunity to easily move among reefs and as such may represent movements that would not occur at more widely spaced reefs.

These results provide new data relevant to planning of reef shark research and management. It is now evident that reef sharks move between closely spaced reefs and undertake large-scale movements. Individuals that moved between reefs and zones spent variable amounts of time within protective areas. Research and management that assumes adult *C. amblyrhynchos* are highly site attached may not produce accurate results. Therefore results of point survey studies must be interpreted with caution. The inter-reef movement observed in larger animals based on telemetry calls into question how much individuals are moving between reefs and how this affects abundance estimates; e.g. [2], [4]. Evidence of higher fidelity in juveniles suggests counts of smaller individuals are more reliable than those for larger individuals, but longer-term acoustic monitoring should be conducted to further examine this. Rapid and repeated movement between reefs and protection zones suggest that small protective zones such as those within the Ribbon Reefs may only be partially successful in sheltering *C. amblyrhynchos* from fishing pressure. This should be considered in future management of these populations.

## Materials and Methods

### Ethics Statement

This research was conducted in accordance with James Cook University animal ethics approval No. A1214.

The Ribbon Reefs (14° 30′ S; 145° 33′ E) in north Queensland, Australia, provide near continuous habitat with individual coral reefs separated by channels of up to 40 m depth and typically 1–1.5 km wide. The study region included six reefs representing six different management zones: Habitat Protection Zone (HP – line fishing, netting and public access permitted), Conservation Park Zone (CP – single line fishing permitted, netting prohibited), Scientific Research Zone (SR – boating and diving permitted, other activities prohibited unless by permit), Marine National Park Zone (MNP – closed to fishing, boating and diving permitted), Preservation Zone (P – no entry except for emergency anchoring and limited research access) and Buffer Zone (B – boating and diving permitted, other activities prohibited unless by permit). Thirty-one VR2 acoustic receivers (Vemco Ltd.) were deployed to passively track movements of *C. amblyrhynchos* from January to July 2008 (Figure 1). Receivers were moored along the reef crest in depths from 10–16 m. Data were downloaded from receivers upon removal from the site in July 2008. Sharks were captured by hook and line fishing, measured, tagged with an external ID tag and surgically implanted with a Vemco RCODE V16 transmitter. Transmitters had a unique pulse series for each shark, operated at 69.0 kHz with randomly spaced transmission intervals from 45–75 s, and a battery life of at least 18 months.

Data from acoustic receivers was processed to define the length of time an individual was monitored (release date to date of last detection), the number of days an individual was present during the monitoring period, and when detections were recorded (day vs night). Data from each reef was examined to define the number of reefs and marine park zones an individual visited and the amount of time spent within each. Significant differences in the proportion of detections of individual sharks between day and night were determined using Chi-squared tests.
